# The Infectious Basis of ACPA-Positive Rheumatoid Arthritis

**DOI:** 10.3389/fmicb.2017.01853

**Published:** 2017-09-27

**Authors:** Lazaros I. Sakkas, Dimitrios Daoussis, Stamatis-Nick Liossis, Dimitrios P. Bogdanos

**Affiliations:** ^1^Department of Rheumatology and Clinical Immunology, Faculty of Medicine, School of Health Sciences, University of Thessaly, Larissa, Greece; ^2^Division of Rheumatology, Department of Internal Medicine, Faculty of Medicine, University of Patras, Patras, Greece

**Keywords:** anti-citrullinated protein antibodies, arthritis, Ebstein-Barr virus, HLA-DRB1 shared epitope, *Porphyromonas gingivalis*

## Abstract

Rheumatoid arthritis (RA) is associated with HLA-DRB1 shared epitope (HLA-DRB1SE) and anti-citrullinated protein autoantibodies (ACPAs). ACPAs precedes the onset of clinical and subclinical RA. There are strong data for three infectious agents as autoimmunity triggers in RA, namely *Porphyromonas gingivalis* and *Aggregatibacter actinomycetemcomitans* causes of periodontal disease (PD), and Epstein-Barr virus (EBV). *P. gingivalis* expresses arginine gingipains, that cleave proteins at the arginine residues, and peptidyl arginine deiminase (PPAD), which citrullinates arginine residues of proteins, thus forming neoantigens that lead to ACPA production. Peripheral blood plasmablasts from ACPA+RA patients produce ACPAs the majority of which react against *P. gingivalis. A. actinocycetemcomitans* produces leukotoxin A, a toxin that forms pores in the neutrophil membranes and leads to citrullination and release of citrullinated autoantigens in the gums. EBV can infect B cells and epithelial cells and resides as latent infection in resting B cells. Abs against citrullinated peptides derived from EBV nuclear antigen appear years before RA and cross-react with human citrullinated fibrin. Citrullinated proteins are potential arthritogenic autoantigens in RA. The conversion of arginine to citrulline increases the peptide binding affinity to HLA-DRB1SE. Also, citrullinated fibrinogen induces arthritis in HLA-DRB1^*^0401 transgenic mice, and transfer of their splenic T cells causes arthritis to recipient mice.

## Introduction

Rheumatoid arthritis (RA) is a systemic inflammatory disease mainly manifested with peripheral polyarthritis. The aetiopathogenesis of the disease is incompletely understood. Risk factors for RA include HLA-DR genes, periodontal disease (PD), and smoking (Bartold et al., 2005; Scher et al., 2012; Mikuls et al., 2014; Kharlamova et al., 2016). The early HLA-DR4 association of RA classified RA by many investigators as an immune-mediated disease and suggested that T cells recognized an antigen presented on HLA-DR4 molecules. The discovery of HLA-DRB1 shared epitope (SE, HLA-DRB1SE), a hypervariable DRβ chain sequence shared by all alleles associated with RA, reinforced this concept (Gregersen et al., 1987; Wordsworth et al., 1989). The discovery of autoantibodies against citrullinated antigens (ACPAs) greatly advanced our understanding of the pathogenetic mechanisms in this disease. ACPAs appear years before clinical onset of RA (Nielen et al., 2004), predict subsequent development of the disease, occur in 50–67% of RA patients, are associated with severe disease, and are highly specific for the disease (van Gaalen et al., 2004; van der Helm-van Mil et al., 2005; Alexiou et al., 2007; Barouta et al., 2017; Hensvold et al., 2017).

Citrullination is a post-translational modification of proteins in which arginine residues are converted to citrulline by the action of enzyme peptidylarginine deiminase (PAD). There are five PAD isoforms (PAD1-4, PAD6), and PAD2 and PAD4 have been implicated in RA. The production of ACPAs means break of tolerance. Tolerance is no immune response to unmodified self. Many proteins are extensively post-translationally modified that including citrullination. In this context, citrullination is a physiological process in many tissues and only in specific circumstances this leads to immune response. Thus, citrullination could create particular neoantigens that would activate T cells, which in turn will provide antigen-specific help to B cells to produce ACPA. Indeed, citrullination increases the affinity of citrullinated antigen to HLA-DRB1SE allele (Hill et al., 2003; Scally et al., 2013). ACPAs in RA recognize many citrullinated autoantigens (Table 1) and are associated with HLA-DRB1SE (Snir et al., 2009), and HLA-DRB1SE appears to be a risk factor for ACPA production in RA rather than an independent risk factor for RA development (van der Helm-van Mil et al., 2006). These findings and the fact that ACPAs are of IgG and IgA class suggest that T cells provide help to B cells for the subsequent ACPA production.

**Table 1:** Examples of citrullinated peptides which are targeted by immune responses against self and non-self immune responses in patients with rheumatoid arthritis.

**Citrullinated protein/peptide**	**Sequence**	**aa**	**References**
Fibrinogen-α chain	GPcitVVEcitHQSACKDS	36-50	Sebbag et al., 2006
Fibrinogen-α chain	VDIDIKIcitSCcitGSCS	171-185	Sebbag et al., 2006
Fibrinogen-α chain	SGIGTLDGFcitHcitHPD	501-515	Sebbag et al., 2006
Fibrinogen-α chain	citGHAKScitPVcitGIHTS	621-635	Sebbag et al., 2006
Fibrinogen-β chain	citPAPPPISGGGYcitAcit	60-74	Sebbag et al., 2006
Enolase-1	KIHAcitEIFDScitGNPTVE	5-21	Lundberg et al., 2008
Vimentin	SAVRAcitSSVPGVR	65-77	Hill et al., 2003
Vimentin	VYATcitSSAVcitLcitSSVP	60-75	Verpoort et al., 2007
Collagen II	AcitGLTGcitPGDA	359-369	Burkhardt et al., 2005
Histone 4	GAKCitHCitKVLCitDNIQGITKPAI	414-34	Corsiero et al., 2016
Histone 4	KPAICitCitLACitCitGGVKCitISGLI	431-50	Corsiero et al., 2016
*P. gingivalis enolase*	KIIGcitEILDScitGNPTVE	5-21	Lundberg et al., 2008
Ebstein-Barr virus EBNA1	GGDNHGCitGCitGCitGCitGCitGGGCitPGAPG	135-58	Pratesi et al., 2006
Ebstein-Barr virus EBNA2	GQSCitGQSCitGCitGCitGCitGCitGCitGKG	338-358	Pratesi et al., 2011

Although smoking is a risk factor for RA (van der Helm-van Mil et al., 2007; Lundberg et al., 2013; Hensvold et al., 2015), and increases citrullination in bronchial tissues (Makrygiannakis et al., 2008), other environmental factors, in addition to smoking, appear to play a predominant role in the development of ACPA+RA (Lee et al., 2007; Hensvold et al., 2015) and infections are likely candidates (Bogdanos and Sakkas, 2017).

## Infections as generators of ACPA in RA

**Figure 1:**
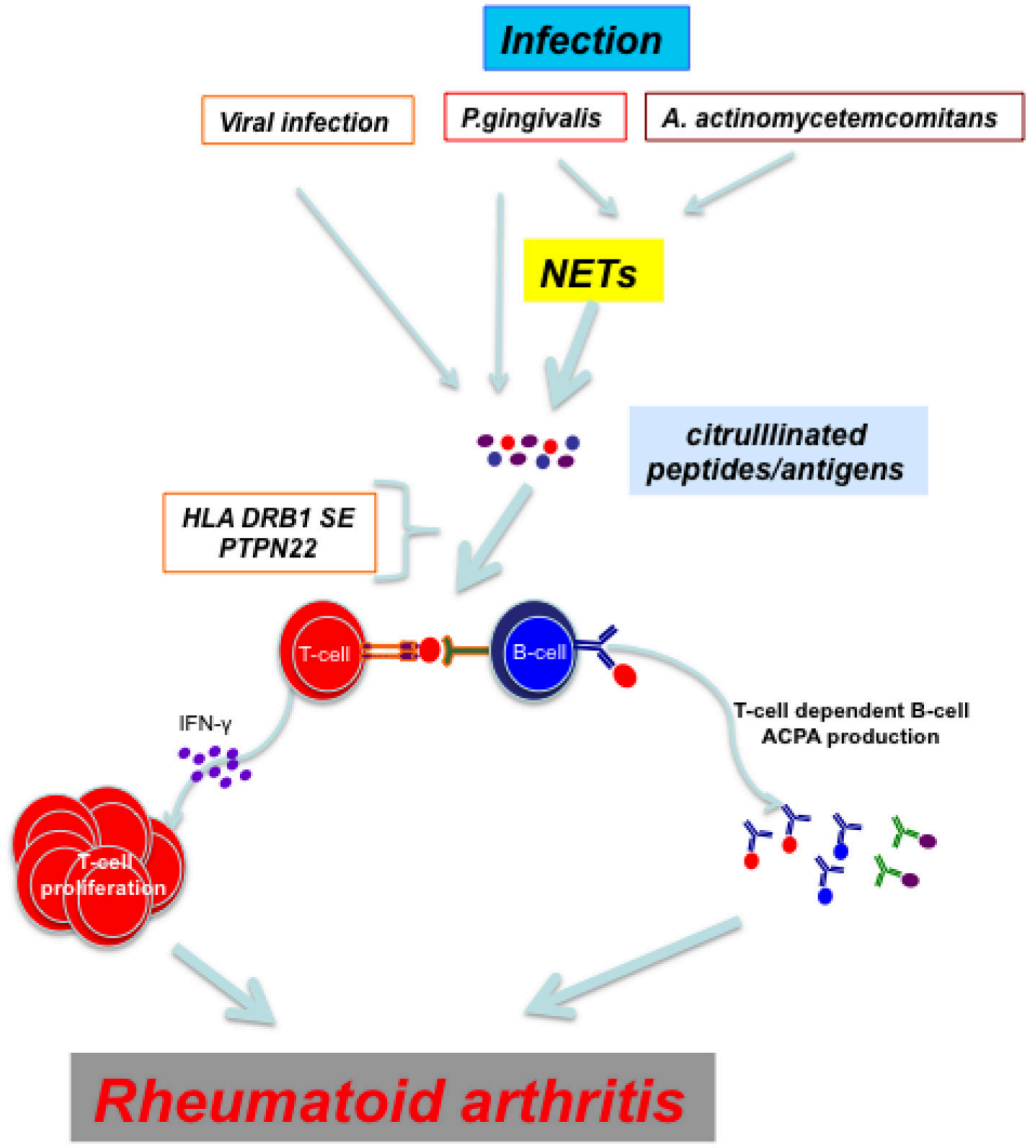
Viral infections and periodontal disease caused by *P. gingivalis* and *A. actinocycetemcomitans* induce directly or through NETs citrullination of proteins/peptides. In an individual with proper genetic background (HLA DRB1 SE and PTPN22 risk allele R620W) T cells recognize citrullinated peptides and mount an immune response which culminates in the development of rheumatoid arthritis.

ACPAs precede the subclinical joint inflammation in pre-RA patients (van de Sande et al., 2011) and can be detected in joints and epithelial sites, such as periodontium in PD (Nesse et al., 2012) and bronchial tissues in early RA (Reynisdottir et al., 2014). Identical citrullinated peptides were found in pulmonary bronchial tissue and synovial membrane and were found to be targets of ACPAs in RA thus providing a link between lungs and joints in ACPA+RA (Ytterberg et al., 2015). A monoclonal ACPA derived from RA patients cross-reacted with many viral, bacterial fungal and plant proteins (Tsuda et al., 2015). The most widely studied infection has been with *P. gingivalis* and Ebstein-Barr virus. The mechanisms by which these infectious agents could trigger RA are illustrated in Figure 1.

### Porphyromonas gingivalis

Chronic PD is very common affecting nearly 30% of adult population (Brown and Loe, 1993) and is caused by various microbes including *Porphyromonas gingivalis* (*P. gingivalis*).

*P. gingivalis* infection, detected by abs against *P. gingivalis* components, have been associated with ACPA in HLA-DRB1SE+RA patients. Anti-*P. gingivalis* abs, detected as abs against RgpB, potent virulent factors of *P. gingivalis* (Haffajee and Socransky, 1994; Kadowaki et al., 1998), showed stronger association with ACPA+RA (Kharlamova et al., 2016). Furthermore, there was additive interaction between these two factors. Anti-RgpB abs also showed more than additive interaction with HLA-DRB1SE in ACPA+RA (Kharlamova et al., 2016). Using anti-*P. gingivalis* lipopolysaccharide abs, one study reported association of anti-*P. gingivalis* abs with ACPA in HLA-DRB1SE+ RA patients and their relatives (Hitchon et al., 2010) whereas another study did not find an association with RA or ACPA status (Seror et al., 2015).

*P. gingivalis* has two unique enzymes, peptidylarginine deiminase (PPAD) and arginine ginpains (Rgps) which are expressed on the bacterial outer membrane and can also be secreted (Potempa et al., 1995; McGraw et al., 1999). Rgps are proteases that cleave proteins at arginine residues, and PPAD citrullinates both bacterial and human proteins (Wegner et al., 2010). *P. gingivalis* PAD citrullinates carboxy-terminal arginine of human proteins following proteolytic cleavage by *P. gingivalis* arginine-gingipains (Wegner et al., 2010). Crystal structure of PPAD and the use of synthetic peptides also revealed that PPAD exhibits a definitive specificity for C-terminal arginine residue created by Rgps, whereas PAD2 and PAD4 preferentially citrullinate internal arginine residues (Goulas et al., 2015; Montgomery et al., 2016). Thus *P. gingivalis* creates neoantigens, not formed by PAD2 and PAD4 and this may explain its pathogenic potential.

It is reasonable to assume that neoantigens, created by Rpgs in conjunction with PPAD in the periodontium of PD, can lead to loss of tolerance and ACPA production. In PD, increased concentrations of anti-CCP and anti-α-enolase autoAbs are detected (Lappin et al., 2013). A peptide 1 of human citrullinated α-enolase (CEP1), an immunodominant epitope, shares 92% homology with *P. gingivalis* α-enolase and cross-reacts with it (Lundberg et al., 2008). This links periodontitis with RA and suggests that periodontal infection can be the inciting agent that breaks immune tolerance in ACPA+RA, although other studies did not find association of PD with RA (Arkema et al., 2010; Eriksson et al., 2016). Using a single-cell ab cloning method, Li et al showed that peripheral blood plasmablasts in ACPA+RA patients produce ACPAs the majority of which cross-react with outer membrane antigens and/or citrullinated a-enolase from *P. gingivalis* (Li et al., 2016).

In addition, *P. gingivalis* can induce neutrophil extracellullar trap (NET) formation (Delbosc et al., 2011), another source of citrullinated autoantigens. NETs are externalized chromatin fibers containing DNA and histones, and decorated with cytoplasmic granular peptides, such as myeloperoxidase, proteinase 3, neutrophil elastase, cathepsin G, LL37, and others, in a process of programmed neutrophil death called NETosis (Yang et al., 2016). PAD4-induced citrullination is an important step in NETosis during which citrullinated histones, vimentin, α-enolase and others are externalized and recognized by ACPAs (Li et al., 2010; Pratesi et al., 2014). PAD4 is also essential for the antibacterial neutrophil immunity (Li et al., 2010). NETosis is enhanced in RA peripheral blood and synovial fluid neutrophils (Khandpur et al., 2013). A positive feedback loop between NETosis and ACPA has been proposed. ACPAs induce NETosis and NETosis provides citullinated autoantigens for ACPA production, as NET components are recognized by RA autoantibodies (Khandpur et al., 2013). Also, B cells from synovial ectopic lymphoid structures (ELS), recognize citrullinated histones of NETs. For instance, monoclonal abs generated from synovial ELS single B cell cloning from patients with ACPA+RA, recognized citrullinated histones from NETs (Corsiero et al., 2016). Neutrophils provide citrullinated autoantigens to the attention of the immune system also via immune-mediated membranolytic pathways (Romero et al., 2013), and this has led to introduction of another pathogen of PD as candidate trigger of autoimmunity in RA, namely *Aggregatibacter actinomycetemcomitans*.

### Aggregatibacter actinomycetemcomitans

*Aggregatibacter actinomycetemcomitans (A.actinomycetemcomitans)* a periodontal pathogen associated with aggressive PD (Haubek and Johansson, 2014) can cause citullination of a broad range of proteins by a completely different mechanism. *A.actinomycetemcomitans* produces leukotoxin A (LtxA), which forms pores on the cell membrane of neutrophils at the crevicular fluid of PD. This leads to neutrophil PAD activation and citrullination of a broad range of proteins, which are released from neutrophils (Konig et al., 2016a). In addition, 47% of RA patients show evidence of *A.actinimycetemcomitans* infection that is associated with ACPA presence. More interestingly, in patients with RA HLA-DRB1SE is associated with ACPA only in patients exposed to *A.actinomycetemcomitans* (Konig et al., 2016a). Thus *A.actinomycetemcomitans* is identified as a strong bacterial candidate triggering autoimmunity in RA.

### Epstein-barr virus

Epstein-Barr virus (EBV) is a herpes virus infecting most of the adult population. EBV can infect B cells and epithelial cells and cause primary infection usually asymptomatic in childhood and then a life-long latent infection in resting memory B cells (Kalla and Hammerschmidt, 2012). High titers of anti-EBV abs are detected in RA patients (Alspaugh et al., 1981), and the EBV DNA load was found to be increased 7–10-fold in PBMCs from RA patients compared to healthy EBV carriers (Balandraud et al., 2003; Lunemann et al., 2008). Furthermore, substantial expansions of CD8+Tcells specific for EBV antigens were detected in PB (Lunemann et al., 2008) and expansions of CD8+T cells reactive against key transactivators of EBV lytic infection were also detected in RA joints (Scotet et al., 1996). Latent membrane protein (LMP) 2A through its immunoreceptor tyrosine activation motif (ITAM) phosphorylates (activates) downstream proteins of B cell receptor thus positively regulating B cell survival and activation (Swanson-Mungerson and Longnecker, 2007).

Abs against citrullinated peptide corresponding to EBV nuclear antigen (EBNA)1 (viral citrullinated peptide 1, VCP1) were detected in RA. Furthermore, affinity-purified anti-VCP1 abs reacted with citrullinated fibrinogen (Pratesi et al., 2006). More importantly, Abs against citrullinated peptides derived from EBNA2 (VCP2) along with abs against histone-4-derived citrullinated peptides appear years before the onset of clinical RA and predict subsequent development of RA (Johansson et al., 2016). These abs were associated with HLA-DRB1SE (Johansson et al., 2016). Abs against VCP1 and VCP2 cross-react with human citrullinated peptides (Pratesi et al., 2011). In particular, competition assays showed that abs to citrullinated peptide EBNA (VCP1) strongly cross-reacted with the citrullinated peptide β60-74 which bears the immunodominant epitope of citrullinated fibrin in RA (Cornillet et al., 2014, 2015). EBV latent transcripts and EBV latent and lytic proteins were detected in germinal center-like ectopic lymphoid structures (ELS) of RA synovial membrane (Croia et al., 2013). Also ACPA producing plasma cells (anti-citrullinated fibrinogen abs) at the periphery of ELS were infected with EBV (expressed lytic proteins). Furthermore, ELS-containing RA synovia transplanted onto severe combined immunodeficiency (SCID) mice produced abs against citrullinated EBV proteins (VCP1 and VCP2) (Croia et al., 2013). These findings provide strong circumstantial evidence that EBV may initiate an immune response which subsequently may be re-directed against self antigens by way of cross-reactivity and epitope spreading.

## Citrullinated proteins as arthritogenic autoantigens

ACPAs are associated with severe disease and are strong predictors of joint erosions in RA (Alexiou et al., 2007; Jilani and Mackworth-Young, 2015). Although association does not prove causation, several lines of evidence suggest that citrullinated proteins are likely to be arthritogenic autoantigens in RA (Sakkas et al., 2014). This means that citrullinated peptides are recognized by and activate T cells, which in turn (a) produce pro-inflammatory mediators and talk to other cells causing joint damage, and (b) provide help to B cells for ACPA production, which by themselves may be pathogenetic.

As already mentioned, the conversion of arginine to citrulline increases the affinity of citrullinated antigen binding to HLA-DRB1SE alleles (Hill et al., 2003; Scally et al., 2013). This has been confirmed in a study by Scally et al who showed that citrulline but no arginine is accommodated within the electropositive P4 pocket of RA-susceptible HLA-DRB1^*^0401/04 alleles (Scally et al., 2013). Furthermore, using HLA-DR4 tetramers, the authors found that citrullinated vimentin- and citrullinated aggrecan-specific T cells were present in peripheral blood of RA patients and their numbers were correlated with disease activity (Scally et al., 2013). Also, a T cell line recognizing citrullinated fibrinogen, abundant in RA joints, induced proinflammatory cytokines, and transfer of this T cell line to mice with CIA exacerbated arthritis (Cordova et al., 2013). Immunization with human fibrinogen (containing citrullinated peptides) in complete Freud adjuvant enhanced arthritis and T cells from these mice were fibrinogen-reactive and produced high levels of IL-6, IFNγ and IL-17 (Ho et al., 2010). Furthermore, adoptive transfer of plasma or T cells caused arthritis in naïve mice (Ho et al., 2010). Citrullinated fibrinogen but not unmodified fibrinogen induced arthritis in HLA-DRB1^*^04:01-IE transgenic mice but not in wild-type C57BL/6 mice (Hill et al., 2008). Furthermore, transfer of splenocytes from these transgenic arthritic mice caused arthritis to recipient mice, indicating that activated citrullinated fibrinogen-specific T cells are crucial for arthritis development (Yue et al., 2010). Also, a pan-PAD inhibitor (Cl-amidine) decreased the clinical severity of collagen-induced arthritis (CIA) and joint and serum protein citrullination (Willis et al., 2011). These studies show that citrullinated peptides in conjuction with HLA-DRB1SE activate T cells which become arthritogenic.

Infection with *P. ginvivalis* further contributes to joint inflammation that is dependent on citrullination. For instance, infection with *P. gingivalis* caused exacerbation of collagen-induced arthritis that was dependent on *P. gingivalis* PAD (PPAD) (Maresz et al., 2013). High levels of citrullinated proteins at the site of infection with *P. gingivalis* were detected as well as ACPAs (Maresz et al., 2013). Also, CIA was much less severe in the presence of PAD-deficient *P. gingivalis* (Gully et al., 2014). On the other hand, *P. gingivalis* components may cause arthritis through molecular mimicry. For instance, immunization of HLA-DR4-IE-transgenic mice with *P. gingivalis* α-enolase either citrullinated or noncitrullinated caused arthritis and abs reactive with human α-enolase (Kinloch et al., 2011). As already mentioned, *P. gingivalis* α-enolase shares sequence similarity with human α-enolase.

ACPAs contribute to joint inflammation and damage since they induce secretion of inflammatory cytokines and differentiation of osteoclasts. ACPA-containing immune complexes induced TNFα secretion by peripheral blood-derived macrophages (Clavel et al., 2008; Laurent et al., 2011; Sokolove et al., 2011), via toll-like receptor 4 (TLR4) and FcγR (Sokolove et al., 2011), whereas citrullinated fibrinogen stimulated TNFα production through TLR4 (England et al., 2017). Also, citrullinated histones increase macrophage TNFα production via TLR4, and immune complexes containing citrullinated histones activate macrophage production of TNFα via TLR4 and FcγR and neutrophils. Over 90% of RA patients have abs against neutrophil-derived citrullinated histones (citrullinated H2B).

*P. gingivalis* may also contribute to arthritis through inflammatory cytokine release. For instance, periodontal disease induced by *P. gingivalis* (Marchesan et al., 2013) or *Prevotella nigrescens* (de Aquino et al., 2014) exacerbated collagen-induced arthritis and promoted Th17 responses.

The effect of citrullinated proteins and ACPAs in wild-type animals (not transgenic animals) in the induction or exacerbation of arthritis is not certain. For instance, immunization of mice with citrullinated histone did not cause arthritis but exacerbated collagen-induced arthritis (Sohn et al., 2015). Similarly, some studies show exacerbating effect of ACPAs on collagen-induced arthritis and others suppressing effects (Kuhn et al., 2006; Shoda et al., 2011; Cantaert et al., 2013). For instance, it has been reported that co-administration of anti-citrullinated fibrinogen abs with anticollagen II abs enhanced CIA (Kuhn et al., 2006).

NETs, apart from providing targets for ACPAs, contribute to inflammatory process in RA. The nuclear and cytoplasmic molecules in NETs have antimicrobial properties, and stimulate adaptive and innate immune responses. LL37 NETs increase fibroblast-like synoviocyte IL-6 and IL-8 production (Khandpur et al., 2013). Also, LL37 can form complexes with DNA and RNA and stimulate innate TLRs (Lande et al., 2007; Ganguly et al., 2009).

ACPAs also contribute to joint erosions. ACPAs and monoclonal ACPAs derived from RA synovial fluid (SF) single B cells enhanced differentiation of osteoclasts through PAD-dependent IL-8 production. Furthermore, transfer of monoclonal ACPAs into mice induced IL-8-mediated bone loss (Krishnamurthy et al., 2016). Also, affinity-purified abs against mutated citrullinated vimentin (MCV) bind to osteoclast surface and induce osteoclastogenesis, whereas adoptive transfer of anti-MCV abs into mice causes osteopenia (Harre et al., 2012).

## Relevance to treatment

Although there may be few disagreements (Konig et al., 2016b), the research data outlined above imply that citrullination, in conjunction with genetic factors, such as HLA-DRB1SE, protein tyrosine phosphatase nonreceptor type 22 (PTPN22) risk allele (Joshua et al., 2016) encoding an R620W amino acid change that allows survival of autoreactive B cells (Menard et al., 2011), is the key element in breaking tolerance. Thus citrullinated peptides may offer new therapeutic strategies for RA. For instance, CTLA-4Ig blocked the development of arthritis induced by citrullinated fibrinogen in HLA-DRB1^*^0401 transgenic mice (Yue et al., 2010). This concept is re-enforced by the study of Gertel et al. who used citrullinate multiepitope peptide derived from prevalent citrullinated autoantigens in RA to reduce disease severity in adjuvant-induced arthritis in rats (Gertel et al., 2015). Also, citrullinated peptide autologous dendritic cells immunotherapy administered to once reduced effector T cells and increased regulatory T cells at 1 month (Benham et al., 2015).

Another strategy could be inhibition of TLR4. TLR4 is an innate immunity receptor for various ligands, including immune complexes containing ACPAs, mainly against citrullinated fibrinogen. Inhibition of TLR4 has been shown to decrease inflammatory arthritis in mouse models (Abdollahi-Roodsaz et al., 2007; Pierer et al., 2011). More importantly, the presence of ACPAs against citrullinated peptides from α-chains and β-chains of fibrinogen and histone 2A in RA patients predicts the anti-inflammatory response of TLR4 inhibition by a therapeutic ab (NI-0101) in an *ex vivo* model of RA (Hatterer et al., 2016). Therefore, it is likely that these new therapeutic strategies will be fruitful in human RA in the near future.

## Author contributions

LS, DD, SL, and DB substantially contributed on drafting the work and revising the manuscript, and approved the final version to be published. LS, DD, SL, and DB agreed to be accountable for all aspects of the work in ensuring that questions related to the accuracy or integrity of any part of the work are appropriately investigated and resolved. LS had the original idea of drafting the manuscript and overall supervision of manuscript's shaping.

### Conflict of interest statement

The authors declare that the research was conducted in the absence of any commercial or financial relationships that could be construed as a potential conflict of interest.
